# Collagen-Based Medical Device as a Stem Cell Carrier for Regenerative Medicine

**DOI:** 10.3390/ijms18102210

**Published:** 2017-10-21

**Authors:** Léa Aubert, Marie Dubus, Hassan Rammal, Camille Bour, Céline Mongaret, Camille Boulagnon-Rombi, Roselyne Garnotel, Céline Schneider, Rachid Rahouadj, Cedric Laurent, Sophie C. Gangloff, Frédéric Velard, Cedric Mauprivez, Halima Kerdjoudj

**Affiliations:** 1 Equipe d’Accueil 4691, Biomatériaux et Inflammation en Site Osseux (BIOS), Pôle Santé, UFR d’Odontologie, SFR-CAP Santé (FED 4231), Université de Reims Champagne-Ardenne, 1 Avenue du Maréchal Juin, 51100 Reims, France; l.aubert@outlook.fr (L.A.); marie.dubus@hotmail.com (M.D.); hkrammal@hotmail.com (H.R.); camille.bour@univ-reims.fr (C.B.); cmongaret@chu-reims.fr (C.M.); sophie.gangloff@univ-reims.fr (S.C.G.); frederic.velard@univ-reims.fr (F.V.); mauprivezcedric@gmail.com (C.M.); 2 UFR de Pharmacie, Université de Reims Champagne-Ardenne, 51100 Reims, France; 3 UFR d’Odontologie, Université de Reims Champagne-Ardenne, 51100 Reims, France; 4 Pole Pharmacie Pharmacovigilance CHU Reims, 51100 Reims, France; 5 Laboratoire d’Anatomie et Cytologie Pathologiques, Centre Hospitalier-Universitaire, Hôpital Robert Debré, 51100 Reims, France; cboulagnon-rombi@chu-reims.fr; 6 CNRS, UMR 7369, Medyc, Université de Reims Champagne-Ardenne, 51100 Reims, France; roselyne.garnotel@univ-reims.fr; 7 Equipe d’Accueil A 3795, Groupe d’Étude des Géomatériaux et Environnement Naturels, Anthropiques et Archéologiques (GEGENAA), Université de Reims Champagne Ardenne, 51100 Reims, France; celine.schneider@univ-reims.fr; 8 CNRS, UMR 7563, LEMTA, Université de Lorraine, 54500 Vandœuvre-Lès-Nancy, France; rachid.rahouadj@univ-lorraine.fr (R.R.); cedric.laurent@univ-lorraine.fr (C.L.)

**Keywords:** stem cell niche, medical device, regenerative medicine, biocompatibility, paracrine activities

## Abstract

Maintenance of mesenchymal stem cells (MSCs) requires a tissue-specific microenvironment (i.e., niche), which is poorly represented by the typical plastic substrate used for two-dimensional growth of MSCs in a tissue culture flask. The objective of this study was to address the potential use of collagen-based medical devices (HEMOCOLLAGENE^®^, Saint-Maur-des-Fossés, France) as mimetic niche for MSCs with the ability to preserve human MSC stemness in vitro. With a chemical composition similar to type I collagen, HEMOCOLLAGENE^®^ foam presented a porous and interconnected structure (>90%) and a relative low elastic modulus of around 60 kPa. Biological studies revealed an apparently inert microenvironment of HEMOCOLLAGENE^®^ foam, where 80% of cultured human MSCs remained viable, adopted a flattened morphology, and maintained their undifferentiated state with basal secretory activity. Thus, three-dimensional HEMOCOLLAGENE^®^ foams present an in vitro model that mimics the MSC niche with the capacity to support viable and quiescent MSCs within a low stiffness collagen I scaffold simulating Wharton’s jelly. These results suggest that haemostatic foam may be a useful and versatile carrier for MSC transplantation for regenerative medicine applications.

## 1. Introduction

In the organism, adult stem cells guarantee the maintenance and repair of tissues and organs. Among them, mesenchymal stem cells (MSCs) are emerging as hopeful candidates for cell-based therapy of numerous diseases (i.e., myocardial infarction, Crohn’s disease, Graft versus host disease, osteoarthritis, etc.) [1,2]. Indeed, along with their differentiation potential and the production of several humoral factors, MSCs are thought to exert regenerative effects by increasing healing rates, modulating inflammation and immune response, promoting angiogenesis, and enhancing tissue remodelling [3].

Adult tissues, such as bone marrow and adipose tissue, have proven to be sources for effective MSCs; however, several disadvantages exist, including availability and invasive and painful procedures required for their isolation. In addition, the health status and the age of the donor may affect MSC expansion capability, function, and/or survival after transplantation [4]. Consequently, scientists are looking for stable, safe, and highly accessible stem cell sources with great potential for regenerative medicine. Since 2004, human MSCs derived from umbilical cord Wharton’s jelly (WJ-MSCs) have become popular in the regenerative medicine community, due to the ease of cell harvesting from scraps of perinatal tissues, which is neither painful nor invasive [5,6]. In addition, their primitive nature sheds light on their significant high proliferation capability and increased in vitro expansion ability when compared to adult MSCs [7].

In the natural environment, stem cells reside in a niche, a microenvironment regulating MSC quiescence, self-renewal, and differentiation. MSCs are isolated from their niche (typically composed of extracellular matrix, niche cells, and secreted stimulants such as growth factors, chemokines, and cytokines), and are traditionally expanded in culture plastic in a two-dimensional (2D) monolayer. However, one of the major limitations in using MSCs ex vivo is that they lose their fundamental properties, such as quiescence and multipotency, limiting their usefulness. The preservation of MSCs in the natural environment is unique, and researchers have been trying to understand how nature maintains the MSCs’ fundamental features.

Advances in material sciences have garnered interest in providing a temporary three-dimensional environment (scaffold) for MSCs, allowing the diffusion of nutrients to ensure cell survival [8]. However, success of these structures is strongly dependent on their structural and mechanical features; ensuring stem cell interactions with the surrounding environment [9,10]. Naturally-derived polymers, such as collagen, are appealing for biological applications due to their enzymatic degradation and proven safety through long-term applications in clinical trials [11].

In light of these thoughts, we address the potential use of collagen-based medical devices (HEMOCOLLAGENE^®^) as WJ-MSCs in vitro niche model. Composition, structure, and mechanical properties of HEMOCOLLAGENE^®^ foam were first investigated before assessment of WJ-MSCs behaviour and morphology once seeded in HEMOCOLLAGENE^®^. The potency of HEMOCOLLAGENE^®^ as an inert WJ-MSCs niche was confirmed through MSC basal cytokines production and maintenance of their undifferentiated state, revealing that HEMOCOLLAGENE^®^ presents suitable properties as a scaffold for WJ-MSCs culture.

## 2. Results and Discussion

**Figure 1:**
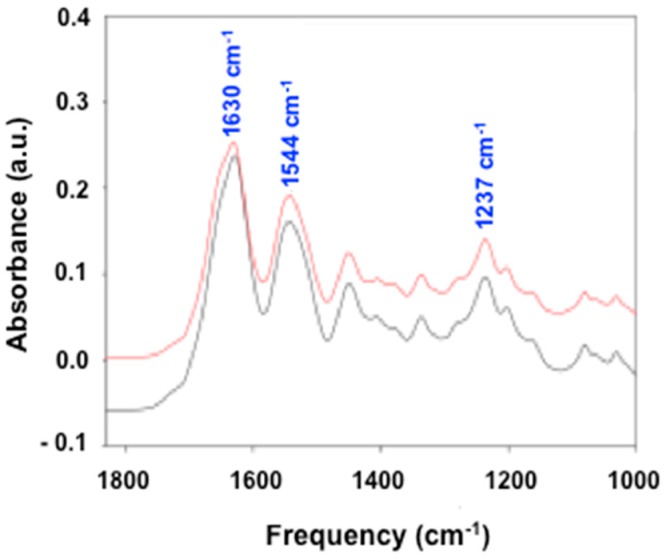
Composition of HEMOCOLLAGENE^®^. Spectra obtained by Fourier transform infrared (FTIR) spectroscopy showing similarities of HEMOCOLLAGENE^®^ foam (red line) with type I collagen (black line).

Collagen-based foams are the most used materials in medical applications. In the present study, we selected bovine collagen foam, manufactured by Septodont (Saint-Maur-des-Fossés, France), as a carrier for stem cell-based therapy. The company´s main product, a foam-like collagen scaffold (HEMOCOLLAGENE^®^), has already been used in dental surgery as a haemostatic device since 1999. HEMOCOLLAGENE^®^ was first characterized in terms of chemical composition. Fourier transform infrared (FTIR) spectroscopy analysis revealed three main peaks at 1237 cm^−1^, 1544 cm^−1^, and 1630 cm^−1^ (Figure 1, red line) characteristics of amide III, II, and I, respectively, matching with the spectra of type I collagen (Figure 1, black line). Additional FTIR spectra attributed to collagen were given in Table S1 in the Supporting Information. A further deep biochemical characterization, performed by ion exchange chromatography, showed that HEMOCOLLAGENE^®^ foam had close similarities with type I collagen with a high amount of glycine, followed by proline, alanine, and hydroxyproline in each specimen (Table 1).

**Figure 2:**
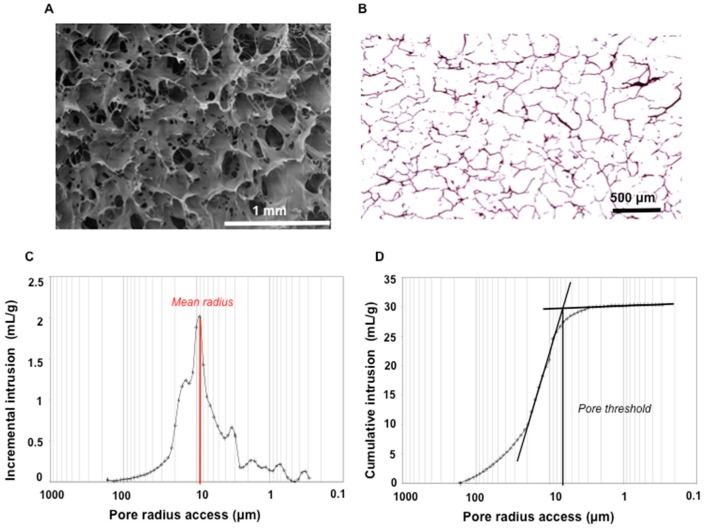
Structure of HEMOCOLLAGENE^®^. (**A**) Representative scanning electron microscopy image (SEM, scale bar indicates 1 mm); (**B**) Hematoxylin-Eosin-Saffron (HES) staining of paraffin-embedded HEMOCOLLAGENE^®^ cross-section (scale bar indicates 500 μm); (**C**,**D**) Pore distribution obtained by mercury intrusion porosimetry showing the mean radius on the incremental curve and the pore threshold on the cumulative curve.

From a structural viewpoint, HEMOCOLLAGENE^®^ exhibited a high interconnectivity and micro/macroporous morphology as depicted in the scanning electron microscopy (SEM) and cross-histological section results (Figure 2A,B). Macro-pore diameters were estimated to be 150 to 500 μm. The total porosity assessed by the mercury intrusion porosimetry method [12] was high, about 95%. The pore access distribution from 165 μm to 3 nm showed a mean radius corresponding to the main peak on Figure 2C of around 9 μm. The pore threshold determined by the intersection of the two tangents of the cumulative intrusion curve was around 6.5 μm (Figure 2D). These values, close to each other, showed that the porous network of HEMOCOLLAGENE^®^ was unimodal and that the pore threshold was significant. To our knowledge, in regenerative medicine applications, porous structures with radii around 150 μm and high porosity (>90%), along with well-interconnected open pores, are required for allowing cell infiltration and efficient nutrient and oxygen diffusion into the structure [8].

**Figure 3:**
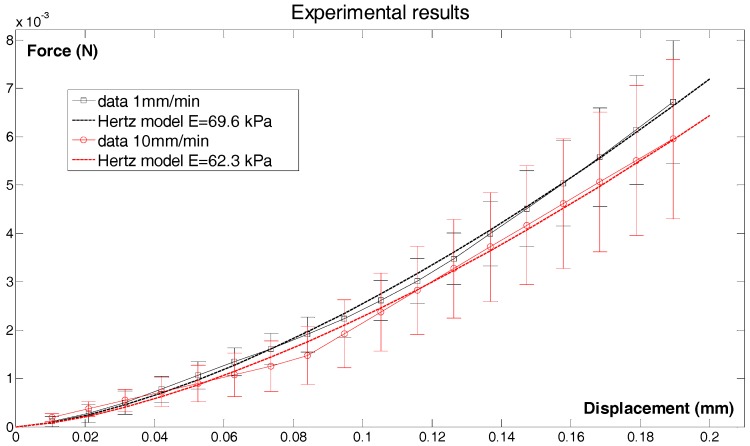
Mechanical features of HEMOCOLLAGENE^®^. Force-displacement responses obtained using indentation tests at 1 mm/min (*n* = 4) and 10 mm/min (*n* = 4). The Young’s modulus has been identified using the Hertz model. No significant difference was observed between the two different testing speeds.

In addition to having suitable architecture, 3D cell carrier foam must exhibit appropriate physical properties to accommodate cell survival. Indentation experiments were used to explore the mechanical features of HEMOCOLLAGENE^®^ foams (Figure 3). The values for the Young’s modulus were 69.6 kPa and 62.3 kPa for foams at indentation rates of 1 mm/min and 10 mm/min, respectively. No significant difference was observed between the identified moduli at the different speeds, indicating no pronounced viscoelastic effect. The obtained values fall well within the values reported in the literature for 3D cell carrier foam [13,14].

The above results highlighted that HEMOCOLLAGENE^®^ foams possess the carrier-required criteria for stem cell based therapy [8]. In the following the results of assays involving the association of HEMOCOLLAGENE^®^ foam with MSCs are presented. A sufficient number of WJ-MSCs from six human umbilical cords were expanded within approximately eight weeks. To reduce heterogeneity of the extracted cell populations [15], WJ-MSCs from three passages with fibroblast-like spindle shapes on culture plastic were used (Figure S1A,B in supporting information section). Moreover, these cells expressed the putative mesenchymal markers CD 44, CD 73, CD 90, and CD 105, but not the hematopoietic markers CD 34, CD 14, and HLA-DR (Figure S1C in the Supplementary Materials). These results, in agreement with previous studies, confirmed their MSC phenotype [16].

**Figure 4:**
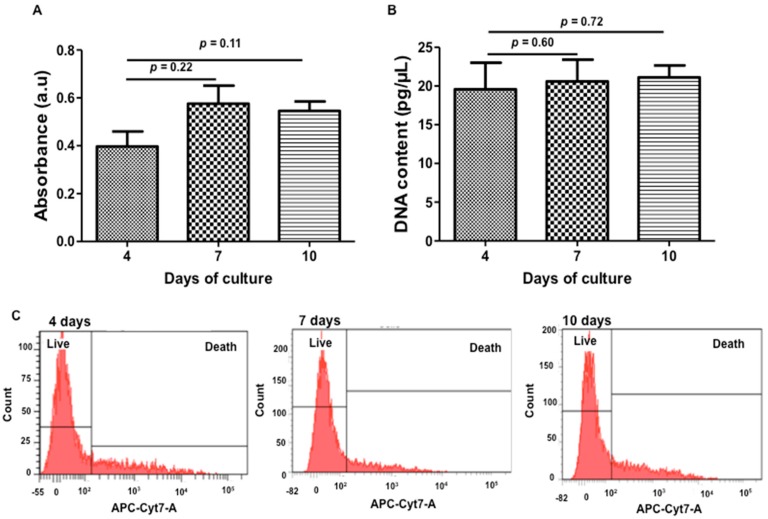
Cell viability over the time of the study. (**A**,**B**): Histogram reflecting WST-1 assay and DNA quantification, respectively; and (**C**): Flow cytometry results obtained after Zombie^®^ labelling. Kinetic study performed after 4, 7, and 10 days of culture showing live and non-proliferating WJ-MSCs cultured within HEMOCOLLAGENE^®^ foam (*n* = 6, Mann and Whitney test).

WJ-MSCs were injected in HEMOCOLLAGENE^®^ foam at density of 8 × 10^4^ cells/foam and placed in 24-well treated-chamber culture to avoid cell migration from foam to plastic. Cytocompatibility of HEMOCOLLAGENE^®^ was firstly monitored by WST-1 (water-soluble tetrazolium salt-1) assay, DNA quantification and Zombie^®^ labelling after 4, 7, and 10 days of culture, using independent foam for each test and time point. While WST-1 and DNA quantification did not show significant variation of measured values, Zombie^®^ labelling revealed a cell survival rate of around 80% over the experimental time (Figure 4).

**Figure 5:**
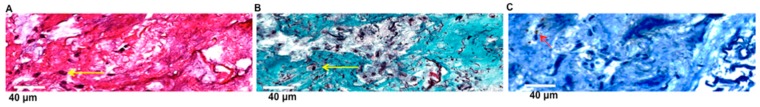
Cell distribution and apoptosis after 10 days of culture. (**A**,**B**) Haematoxylin-eosin-saffron (HES) and Masson’s trichrome staining of paraffin-embedded cellularized HEMOCOLLAGENE^®^ cross-sections, respectively. HES staining showing nuclei in blue (yellow arrow) and collagen in orange. Masson’s trichrome staining, showing collagen in green and nuclei in brown (yellow arrow); and (**C**) cleaved caspase-3 immunohistochemistry showing few apoptotic cells (red arrow) within HEMOCOLLAGENE^®^. (Scale bars indicate 40 μm).

These results indicated that HEMOCOLLAGENE^®^ foams are a suitable environment for stem cell culture. It is generally accepted that an increase in the WST-1 optical density at 450 nm and in DNA values reflects a proliferating state of cells; in our study, the number of WJ-MSCs in HEMOCOLLAGENE^®^ foam remained stable over the culture time, a signature of the non-proliferating state. Moreover, the high survival rate reflected non-toxic culture conditions; meeting the basic requirements of tissue engineering scaffolds [11]. In addition to cell survival, a uniformity of cell distribution within scaffolds is required for 3D culture systems. A general WJ-MSCs distribution within HEMOCOLLAGENE^®^ up to 10 days of culture was given by HES and Masson’s trichrome staining (Figure 5). Histological cross-sections showed randomly-distributed WJ-MSCs embedded within the fibrous extracellular matrix (Figure 5A,B, yellow arrow). Few apoptotic WJ-MSCs revealed by cleaved caspase-3 immunohistochemistry were detected in the inner region of the foam (Figure 5C, red arrow), strengthening the Zombie^®^ results. Finally, we noticed the absence of squamous cells within the HEMOCOLLAGENE^®^ foam, the absence of glycosaminoglycane synthesis, and the absence of mineralization nodules, signature of the absence of spontaneous cell differentiation into adipose, cartilaginous and bone lineage, respectively.

**Figure 6:**
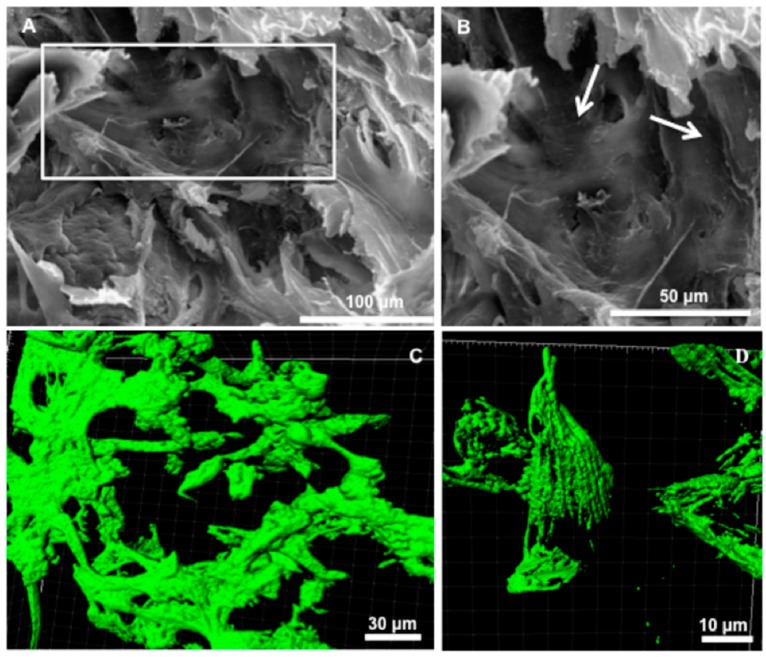
Cell morphology after 10 days of culture. (**A**,**B**) Over and closer (rectangle) scanning electron microscopy views (scale bars indicate 100 and 50 μm, respectively), highlighting cell distribution within pores (white arrows); (**C**,**D**) Over and closer Imaris 3D views of cytoskeleton-labelled cells (scale bars indicate 30 and 10 μm, respectively), showing actin bundles and highly-elongated cell morphology.

The low percentage of apoptotic/necrotic cells within foam might be attributed to the preservation of porosity and pore interconnectivity over the culture time, allowing mass transfer exchanges between foam and culture medium (i.e., nutriment diffusion and cell metabolite release). Furthermore, pore size and pore interconnectivity influence cell shape. Cells residing in open-pore scaffold exhibit an elongated shape in contrast to closed-pore scaffold where cells adopt a rounded morphology [8,17]. A deeper investigation of WJ-MSCs morphology within HEMOCOLLAGENE^®^ foam was performed by SEM and CLSM experiments. SEM imaging showed that WJ-MSCs adopted flattened morphology within HEMOCOLLAGENE^®^ pores (Figure 6). These observations were further supported by CLSM micrographs of Phalloidin^®^ stained cytoskeleton, showing actin bundles of aligned long filaments and highly elongated morphology. These results clearly demonstrate that WJ-MSCs establish physical interactions with the HEMOCOLLAGENE^®^ foam.

Cell spreading is a complicated process, which, besides biophysical cues (mechanical and structure), is also associated with the chemical composition of materials [8]. MSCs have been shown to possess a strong attachment to type I collagen through arginine-glycine-aspartate (RGD) binding domains with the cell membrane integrins [18,19]. Gathering HEMOCOLLAGENE^®^ composition and porosity (Figure 1 and Figure 2), the expected high cellular bond is confirmed. During experimental studies, we noticed that WJ-MSC-loaded HEMOCOLLAGENE^®^ foams shrank drastically (Figure S2), which could be due to their enzymatic degradation. However, the lack of significant variation of the hydroxyproline rate in the supernatant (12, 16, 18, and 21 μM, after 1, 4, 7, and 10 days of culture, respectively, versus 12,680 μM for totally hydrolyzed foam), suggests a possible mechanical instability of HEMOCOLLAGENE^®^ foams or active contractile forces generated by WJ-MSCs [20].

Mesenchymal stem cells within niches respond to biochemical, structural, and mechanical cues from their surrounding microenvironment, affecting their constitutive cytokines secretion/self-renewal/differentiation, thus contributing to tissue homeostasis [21]. The present study was designed to determine if WJ-MSCs cultured with HEMOCOLLAGENE^®^ foams maintain a constitutive secretory profile. Therefore, their ability to regulate and release cytokines, over the experimental time, such as IL-6, IL-8, IL-10, and vascular endothelial growth factor (VEGF), was followed at mRNA (i.e., *IL6*, *CXCL8*, *IL10*, and *VEGFA*) and protein levels by qRT-PCR and ELISA, respectively.

**Figure 7:**
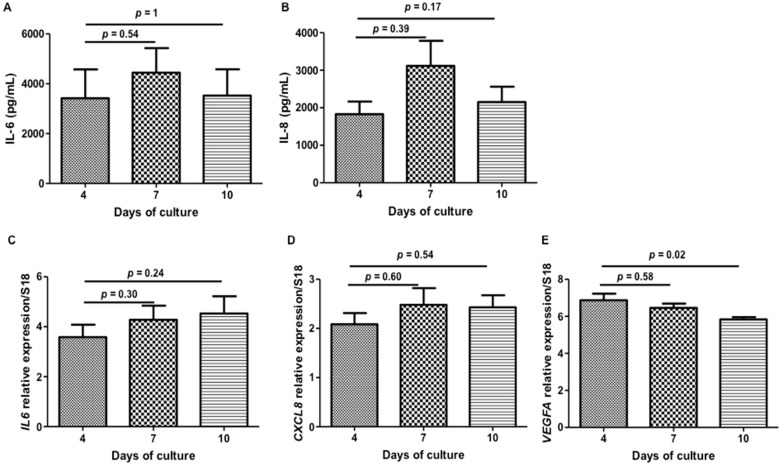
Cell paracrine activity over the time of the study. (**A**,**B**) Histograms of IL-6 and IL-8 ELISA quantification, respectively, showing non-significant production of related proteins; (**C**–**E**) Histograms of *IL6*, *CXCL8*, and *VEGFA* qRT-PCR analysis, showing non-significant regulation of *IL6* and *CXCL8*, and significant *VEGFA* down-regulation (*n* = 6, Mann and Whitney statistical test, *p* < 0.05 for four versus 10 days of culture).

Among quantified cytokines, only IL-6 and IL-8 were detected in supernatants, whereas no regulation of *IL6* and *CXCL8* was noticed, suggesting a constitutive secretion of these cytokines over the kinetic study (Figure 7). While IL-6 and IL-8 releases were constant over the experimental time, we were able to distinguish a slight increase at day 7 (Figure 7A–D). Related to an anti-inflammatory profile, neither *IL10* regulation nor IL-10 release in supernatant were detected, probably due to the absence of a pro-inflammatory environment stimuli [22]. Despite a significant down-regulation of *VEGFA* at day 10, released VEGF was not detected in the culture supernatant over the time (Figure 7E), suggesting that VEGF production is under the detection threshold of the kit or VEGF is accumulated within the cell cytoplasm. Finally, as bone is composed of type I collagen, we looked for the BMP-2 release in the medium, which was not detected in the supernatant over the study time.

**Figure 8:**
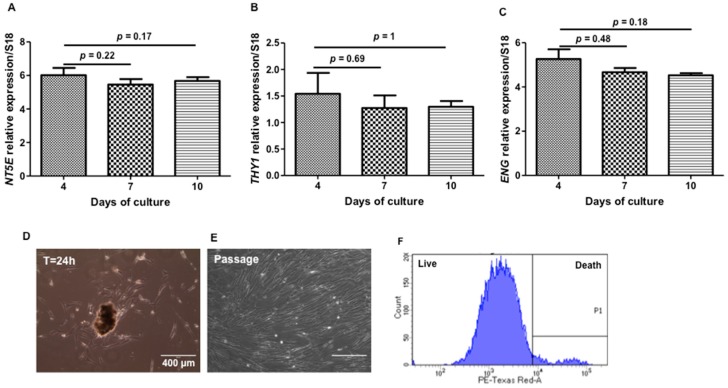
Cell phenotype over the time of the study. (**A**–**C**) Histograms of *NT5E* (CD 73), *THY1* (CD 90), and *ENG* (CD 105) qRT-PCR analysis, respectively, showing non-significant regulation of MSCs markers; (**D**) WJ-MSC migration on plastic; (**E**) WJ-MSC morphology after passage; and (**F**) flow cytometry results obtained after Zombie^®^ labelling of amplified cells (*n* = 6, Mann and Whitney statistical test).

IL-6 and IL-8 are currently described as pro-inflammatory cytokines, but in our experiments, their basal level secretion suggests that HEMOCOLLAGENE^®^ represents an “inert” environment for WJ-MSCs and cell supports secretory function. While the basic biological role of IL-8 is attracting and activating neutrophils, Boyden migration assays did not show significant increase in the recruitment of neutrophils by WJ-MSC loaded-HEMOCOLLAGENE^®^ conditioned media compared to cell-free HEMOCOLLAGENE^®^ conditioned media (Figure S3), confirming both the neutral role of HEMOCOLLAGENE^®^ foam and constitutive IL-8 cytokine production of WJ-MSCs cultured in HEMOCOLLAGENE^®^. IL-6 cytokine is described to maintain the “stemness” of MSCs [23,24]. Therefore, the expression of NT5E, THY1 and ENG (genes corresponding to CD 73, CD 90, and CD 105, respectively) was followed by qRT-PCR after 4, 7, and 10 days of culture. With respect to stem cell associated markers, WJ-MSCs cultured in HEMOCOLLAGENE^®^ did not show any gene variations over the time (Figure 8A–C), suggesting a role of HEMOCOLLAGENE^®^ in maintaining WJ-MSCs under the undifferentiated state. Furthermore, once placed on plastic culture, WJ-MSCs were able to migrate and proliferate without any cell damage and spontaneous differentiation (Figure 8D–F).

## 3. Materials and Methods

### 3.1. Materials

A haemostatic medical device named HEMOCOLLAGENE^®^ foam was provided from Septodont, France. According to the manufacturer, HEMOCOLLAGENE^®^ is made of bovine non-denatured collagen and obtained by a freeze-dried process.

#### 3.1.1. Scanning Electron Microscopy (SEM)

Foam morphology was investigated by SEM with a LaB6 electron microscope (JEOL JSM-5400LV), on sputter-coated foam with thin gold–palladium film (JEOL ion sputter JFC 1100, Croissy Sur Seine, France). Images were acquired from secondary electrons at a primary beam energy of 10 kV.

#### 3.1.2. Fourier Transform Infrared (FTIR)

FTIR spectra were obtained by a Fourier transform infrared-attenuated total reflection (FTIR-ATR, Vertex 70 spectrometer, Bruker, Ettlingen, Germany) using a DTGS detector. Type I collagen from bovine (medical grade; Symatese, Lyon, France) was used as control.

#### 3.1.3. Ion Exchange Chromatography

Amino acid composition of HEMOCOLLAGENE^®^ foam was determined by ion exchange chromatography (HITACHI 8800 analyzer, Science Tec, Tokyo, Japan). One milligram of foam was hydrolysed in HCl 6 M at 110 °C for 18 h. Dried hydrolysates obtained under nitrogen stream evaporation were then resuspended in 100 µL of a buffer composed of lithium 13.86 mM, lithium citrate 55 mM, citric acid 207 mM, ethanol 6% (*v/v*), and thiodiglycol 1% (*v/v*) pH 2.8. Type I collagen from bovine (medical grade; Symatese) was used as control. The chromatography was performed according to the manufacturer’s instructions. Amino acid composition was expressed as residue numbers per 1000.

#### 3.1.4. Mercury Intrusion Porosimetry

Pore access radii distribution and the total porosity of HEMOCOLLAGENE^®^ foam were assessed by mercury intrusion porosimetry (Micromeretics AutoPore IV 9500, Hexton, UK). The measured pore access radius ranges from 165 µm (0.005 MPa) to 0.003 µm (274 MPa). Thus, pores larger or thinner than these sizes are not considered by this technique. The incremental curve gives the mean pore radius for which the intrusive volume is maximal. The cumulative curve allows plotting the pore threshold that corresponds to the pore access allowing filling of the main part of the porous network. When both radii are close, the pore distribution can be considered as unimodal.

#### 3.1.5. Micro-Indentation

Mechanical properties were explored by a custom micro-indentation setup (LEMTA), as proposed in the literature [25]. HEMOCOLLAGENE^®^ foam was placed on an electrical balance used as a force sensor, and compressed with a spherical indenter (radius *r* = 0.75 mm). Indentation speeds of 1 mm/min (*n* = 4) and 10 mm/min (*n* = 4) were used.

**Figure 9:**
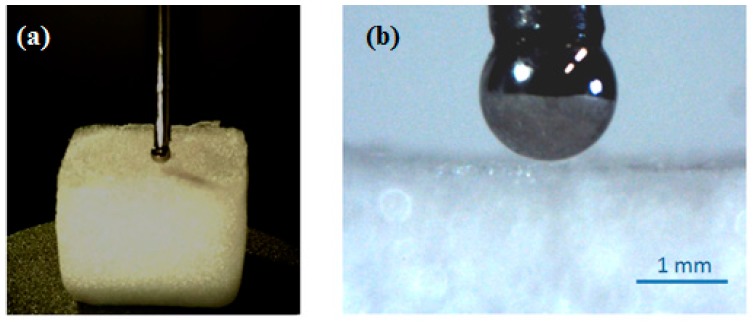
Micro-indentation tests performed on HEMOCOLLAGENE^®^ foam. (**a**) Global view of the indentation test on a typical specimen (10 × 10 × 10 mm); (**b**) Zoom of the spherical indenter (radius = 0.75 mm).

The force applied to the foam was then measured as a function of the applied displacement *u* of the spherical indenter (Figure 9). A simple Hertz contact model was used to fit with the experimental force-displacement curves using a least-square method, and the Young’s modulus *E* of the foam was identified with this model:

$$F=\frac{16}{9}E\;r^{\frac{1}{2}}\;u^{\frac{3}{2}}$$

### 3.2. Biological Experiments

#### 3.2.1. Cell Culture

Mesenchymal stem cells were enzymatically isolated from fresh human umbilical cords obtained after full-term births as previously described [5] and amplified at a density of 3 × 10^3^ cell/cm^2^ in α-MEM culture medium (Lonza, Saint Quentin, France) supplemented with 10% decomplemented FBS, 1% Penicillin/Streptomycin/Amphotericin B, and 1% Glutamax (*v*/*v*, Gibco, Villebon-sur-Yvette, France), and maintained in a humidified atmosphere of 5% CO_2_ at 37 °C with a medium change every three days. Human umbilical cord harvesting was approved ethically and methodologically by our local research institution and was conducted with informed patients (written consent, non-opposition) in accordance with the usual ethical legal regulations (Article R 1243-57). All procedures were done in accordance with our authorization and registration number DC-2014-2262 given by the National “Cellule de Bioéthique”. At third passage, Wharton’s jelly mesenchymal stem cells (WJ-MSCs) were characterized by flow cytometry (FACSCalibur; BD Bioscience, le Pont de Claix, France) through the expression of CD 73, CD 90, CD 44, CD 105, CD 34, CD 14, and HLA-DR (BD, le Pont de Claix, France), Table S2 in Supplementary Materials) and then used in our experimental procedure at the fourth passage.

WJ-MSCs suspension (8 × 10^4^ cells) were injected with a 1 mL syringe and through a 21 G needle, in the middle of HEMOCOLLAGENE^®^ foam. After 4 h of incubation at 37 °C, WJ-MSCs loaded HEMOCOLLAGENE^®^ foams were transferred to 24-well coated plates preventing any cell migration and adhesion (Nunclon Sphera, Thermo scientific, Villebon-sur-Yvette, France). WJ-MSC-loaded HEMOCOLLAGENE^®^ foam was cultured in 1 mL of α-MEM culture medium and changed after 1, 4, 7, and 10 days of culture. Supernatants after 4, 7, and 10 days were collected, centrifuged at 250× *g*, and conserved at −20 °C (conditioned media).

#### 3.2.2. WST-1

Mitochondrial activity, followed by WST-1 cell proliferation assay (Roche Diagnostics, Meylan , France), was performed after 4, 7, and 10 days of WJ-MSCs culture in HEMOCOLLAGENE^®^ foam in accordance with the manufacturer protocol. Absorbance was measured at 440 nm using a FLUOstar Omega microplate reader (BMG Labtech, Champigny-sur-Marne, France) against a background control as blank. A wavelength of 750 nm was used as the reference wavelength.

#### 3.2.3. DNA Quantification

DNA quantification was performed on extracted DNA after 4, 7, and 10 days of WJ-MSCs culture in HEMOCOLLAGENE^®^ foam, using a MasterPure^TM^ DNA Purification Kit (Epicentre, Biotechnologies, Strasbourg, France) in accordance with the manufacturer’s protocol. The quantity of extracted DNA was assessed by measuring the absorbance at 260 nm (Nanodrop, Thermo Scientific, Villebon-sur-Yvette, France) and the 260/280 nm absorbance ratio for all measured samples was between 1.8 and 2.

#### 3.2.4. Zombie^®^ Labeling

Cell membrane integrity was assessed by Zombie^®^ labelling (Ozyme, Montigny-le-Bretonneux, France) in accordance with the manufacturer’s protocol. Labelled cells within HEMOCOLLAGENE^®^ foams were released using collagenase treatment for 5 min, fixed with 4% (*w*/*v*) paraformaldehyde (Sigma-Aldrich, Guyancourt, France), and analysed by an LSRFortessa flow cytometer (BD Bioscience, France).

#### 3.2.5. SEM

SEM was performed after 10 days of culture on fixed WJ-MSCs loaded HEMOCOLLAGENE^®^ foam with 2.5% (*w*/*v*) glutaraldehyde (Sigma-Aldrich) at room temperature for 1 h. Samples were dehydrated in graded ethanol solutions from 50% to 100% and in hexamethyldisilazane (Sigma, France) for 10 min. After air-drying at room temperature, samples were immersed in liquid nitrogen, fractured, then sputtered with a thin gold–palladium film under a JEOL JFC 1100 ion sputter and viewed using a LaB6 electron microscope (JEOL JSM-5400 LV, France). Images were acquired from secondary electrons at a primary beam energy of 20 kV.

#### 3.2.6. Confocal Laser Scanning Microscopy (CLSM)

Confocal laser scanning microscopy (CLSM) was performed for visualization of Phalloidin^®^-labelled cytoskeletons after 10 days of culture of WJ-MSCs loaded HEMOCOLLAGENE^®^ foam. Samples were fixed in 4% (*w*/*v*) paraformaldehyde (Sigma-Aldrich, France) at 37 °C for 10 min and permeabilized with 0.5% (*v*/*v*) Triton X-100 for 5 min. Then, Phalloidin^®^ coupled to AlexaFluor^®^ 488 (Invitrogen, 1:100 dilution in 0.1% Triton X-100) was incubated for 30 min at room temperature, rinsed twice, and labelled cytoskeletons were imaged by CLSM (Zeiss microscopy, Oberkochen, Germany, objectives × 20 and × 63).

#### 3.2.7. Histology and Immunohistochemistry

Cell distribution and apoptotic cells were assessed by histology and immunohistochemistry, respectively. After 10 days of culture, WJ-MSCs loaded HEMOCOLLAGENE^®^ foams were fixed in 4% (*w*/*v*) paraformaldehyde for 1h and paraffin embedded after ethanol dehydration using a Shandon Excelsior Tissue Processor (Thermo Fisher Scientific, Waltham, MA, USA). Five micrometer thick sections were performed on paraffin-embedded samples (rotation microtome AP280, Leica Microsystems). Hematoxylin-eosin-saffron (HES) and Masson’s trichrome staining were performed separately on consecutive tissue sections and images were taken using a scanner iScan Coreo AU (Roche Ò Ventana). For immunohistochemistry, after deparaffinization, sections were incubated with the Cell Conditioner 1 (EDTA, pH 8.4) for 64 min, followed by preprimary peroxidase inhibition and incubation with the primary antibody anti-cleaved Caspase-3 (rabbit polyclonal, Cell Signaling Technology, Danvers, MA, USA) at a 1:600 dilution at 37 °C for 32 min on the automated staining instrument BenchMark XT (Ventana Medical System). Then, the staining reaction was performed using the UltraView Universal DAB v3 Kit (Ventana Medical System, Tucson, AZ, USA). The counterstain and post-counter-stain comprised haematoxylin and bluing reagent. Images were taken using an iScan Coreo AU scanner.

#### 3.2.8. ELISA Quantification

Cytokine and growth factor releases were assessed by ELISA. The quantification of IL-6, IL-8, IL-10, and VEGF proteins at 4, 7, and 10 days in conditioned supernatants was assessed using respectively human IL-6, IL-8, IL-10 and human VEGF Duoset^®^ (R&D systems, Lille, France). Absorbance was measured at 450 nm with correction of non-specific background at 570 nm according to the manufacturer’s instructions.

#### 3.2.9. Chemotaxis Assay

Neutrophil migration assay was followed by Boyden chamber chemotaxis assay. Neutrophils were collected as previously described [26]. Conditioned media from WJ-MSCs loaded Hemocollagene^®^ foam cultured for 4, 7, and 10 days were deposited on the lower compartment, whereas 5 × 10^4^ neutrophils were seeded on a polycarbonate membrane (5 μm pores, Nucleopore Track-etch membrane, Whatman, Maidstone, UK) in the upper compartment. After 45 min of incubation at 37 °C in 5% CO_2_, non-migrating neutrophils were removed from the top of the membrane and migrated cells at the bottom were stained with May-Grünwald Giemsa (RAL555 kit) and imaged (Axiovert 200M microscope, Zeiss, Oberkochen, Germany, Objective × 40). Conditioned media from cell-free Hemocollagen^®^ foam cultured for 4, 7, and 10 days were used as control.

#### 3.2.10. Quantitative Real Time Polymerase Chain Reaction (qRT-PCR)

Mesenchymal markers, cytokines, and growth factor gene expressions were assessed by qRT-PCR. After 4, 7, and 10 days of culture in Hemocollagene^®^ foams, total RNAs of WJ-MSCs were extracted using MasterPureTM RNA Purification Kit (Epicentre^®^ Biotechnologies, Strasbourg, France) in accordance with the manufacturer protocol. RNA purity was assessed by measuring the absorbance ratio at 260/280 nm (Nanodrop 2000C, Thermo Scientific, France), which was comprised between 1.8 and 2. Total RNAs (500 ng) were reverse transcribed into cDNA using a high-capacity cDNA reverse transcription kit (Applied Biosystems, Villebon-sur-Yvette, France) following the manufacturer instructions. Ten nanograms of reverse transcription product were amplified by qRT-PCR on a StepOne Plus TM system (Applied Biosystems, Villebon-sur-Yvette, France). Using this approach, the transcriptional levels of *RPS18* (internal control), *NT5E*, *THY1*, and *ENG* (corresponding to CD73, CD90, CD105 MSC markers, respectively), *IL6*, *CXCL8*, *IL10* (corresponding to IL-6, IL-8, IL-10 cytokines, respectively), and *VEGFA* (vascular endothelial growth factor) were determined using the double strand-specific Power SYBR^®^ Green dye system (Applied Biosystems, Villebon-sur-Yvette, France). After a first denaturation step at 95 °C for 10 min, qRT-PCR reactions were performed according to a thermal profile that corresponds to 40 cycles of denaturation at 95 °C for 15 s, annealing and extension at 60 °C for 1 min. Data collection was performed at the end of each annealing/extension step. The third step that consists in a dissociation process is performed to ensure the specificity of the amplicons by measuring their melting temperature (Tm). Data analysis was performed with the StepOneTM Software v2.3 (Applied Biosystems, Villebon-sur-Yvette, France).

### 3.3. Statistical Analysis

All the results were obtained with six independent umbilical cords. Results were represented on histograms as mean ± standard error of the mean using GraphPad Prism version 5.00 for Windows, (GraphPad Software, San Diego, CA USA, www.graphpad.com). All statistical analyses were performed using GraphPad Prism version 5.00 and, for Mann and Whitney tests, a value of *p* < 0.05 was accepted as statistically significant (rejection level of the null-hypothesis of equal means).

## 4. Conclusions

The mesenchymal stem cell niche represents a microenvironment regulating MSCs’ quiescence, self-renewal, and differentiation. Thus, an ideal scaffold would keep MSCs under an undifferentiated state until host-derived paracrine signals induce their activation and commitment. In this work, the association of a dense and porous clinical haemostatic device with human MSCs was proposed as a versatile in vitro MSCs niche presenting suitable intrinsic properties (i.e., composition, porosity, and elastic modulus) for human MSC cultures. Biological investigations revealed an apparently inert microenvironment of HEMOCOLLAGENE^®^ foam, where cells are able to remain viable, in an undifferentiated state with basal cytokine secretion.

MSCs are used in regenerative medicine in two contexts: autologous and allogeneic [27,28]. Integration of autologous MSCs into the damaged tissues is sought, whereas a long-term integration of allogeneic MSCs is not expected, even if MSCs express only low levels of the histocompatibility markers. Their paracrine activity along with activation of resident stem cells and mobilization of circulating ones into the damaged tissues are, thus, relevant [29]. In view of application of HEMOCOLLAGENE^®^ as a useful WJ-MSC carrier in the regenerative medicine field, additional studies under in vitro and in vivo mimicking pathological conditions (i.e., hypoxic and/or inflammatory stimuli) are required to identify whether host derived paracrine signals could stimulate regenerative properties of WJ-MSCs loaded in HEMOCOLLAGENE^®^.

## Figures and Tables

**Table 1:** Amino acid content assessed by ion exchange chromatography (residue numbers/1000).

Amino-Acid	HEMOCOLLAGENE^®^	Type I Collagen (Reference)
Hydroxyproline	91.2	101.4
Aspartic acid	49.7	49.7
Threonine	19.4	18.9
Serine	33.7	28.8
Glutamic acid	75.2	72.1
Proline	126.8	117.3
Glycine	322.5	327.5
Alanine	106.5	114.8
Valine	23.8	20.9
Methionine	3.1	7.5
Isoleucine	13.5	11.9
Leucine	26.9	25.8
Tyrosine	2.0	2.0
Phenylalanine	14.7	13.9
Hydroxylysine	6.3	6.5
Lysine	24.6	26.3
Histidine	5.3	5
Arginine	54.6	49.7

## Supplementary Materials

Supplementary materials can be found at www.mdpi.com/1422-0067/18/10/2210/s1.

## Abbreviations

3D	Three-Dimensional
MSCs	Mesenchymal Stem Cells
WJ-MSCs	Wharton Jelly-Derived Mesenchymal Stem Cells
IL	Interleukin
SEM	Scanning Electron Microscopy
CLSM	Confocal Laser Scanning Microscopy
FTIR	Fourier Transform Infrared spectra
CD	Cluster of Differentiation
qRT-PCR	Quantitative Real Time Polymerase Chain Reaction
HES	Hematoxylin-Eosin-Saffron
WST-1	Water-Soluble Tetrazolium Salt-1
DNA	Deoxyribonucleic Acid
VEGF	Vascular Endothelial Growth Factor
ELISA	Enzyme-Linked Immunosorbent Assay
