# Supplementary material for: Collagen-Based Medical Device as a Stem Cell Carrier for Regenerative Medicine
Source: Int J Mol Sci. 2017 Oct 21;18(10):2210. doi: 10.3390/ijms18102210 (PMC5666890; doi:10.3390/ijms18102210)
Supplement: Supplementary file 1 [file ijms-18-02210-s001.pdf]

International Journal of *Molecular Sciences*

MDPI

Supplementary Materials: Collagen-Based Medical Device as a Stem Cell Carrier for Regenerative Medicine

Léa Aubert, Marie Dubus, Hassan Rammal, Camille Bour, Céline Mongaret, Camille Boulagnon-Rombi, Roselyne Garnotel, Céline Schneider, Rachid Rahouadj, Cedric Laurent, Sophie C. Gangloff, Frédéric Velard, Cedric Mauprivez and Halima Kerdjoudj

Table S1. FTIR vibration bands of HEMOCOLLAGENE® foam.

Wavenumbers (cm^{-1})	Assignment
1630	ν(C=O)
1544	ν(C–N), (N–H)
1454	δ(CH_2), δ(CH_3)
1403	δ(CH_2), δ(CH_3)
1340	δ(CH_2), δ(CH_3)
1282	ν(C–N), δ(N–H)
1237	ν(C–N), δ(N–H)
1203	ν(C–N), δ(N–H)
1160	ν(C–O)
1079	ν(C–O), ν(C–O–C)
1035	ν (C–O), ν(C–O–C)

Table S2. Table summarizing antibodies features used in cytometry flow experiments (BD, France).

Anti-Bodies	Clone	Host	Catalog Number	Fluorochrom	Isotype
CD14	M5E2	Mouse	555397		IgG2a, κ
CD34	581	Mouse	555821	FITC	IgG1, κ
CD44	G44-26	Mouse	555478	FITC	IgG2b, κ
CD73	AD2	Mouse	550257	PE	IgG1, κ
CD90	5E10	Mouse	555596	PE	BALB/c IgG1, κ
CD105	266	Mouse	560839	PE	BALB/c IgG1
HLA-DR	G46-6	Mouse	555811	FITC	IgG2a, κ

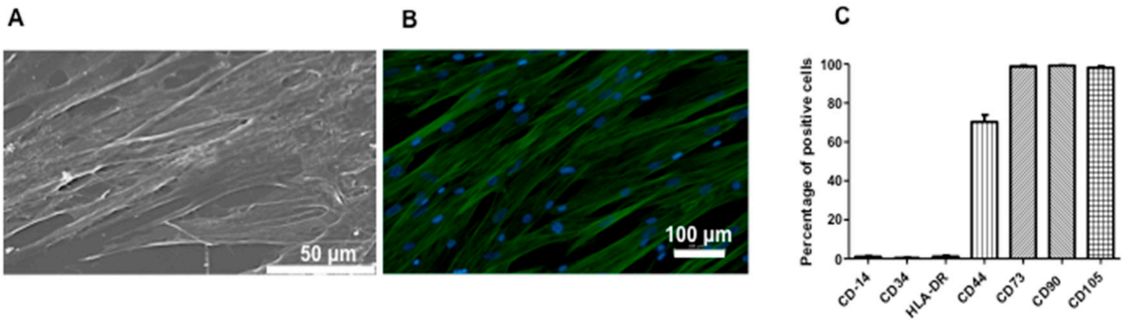

Figure S1. Characterization of human Wharton's jelly derived mesenchymal stem cells. (**A**) scanning electron microscopy and (**B**) cytoskeleton labelled cells showing elongated cells (scale bars indicate 50 and 100 µm, respectively); (**C**) Flow cytometry results indicating CD 14, CD 34 and HLA-DR negative cells and CD 44, CD 73, CD 90 and CD 105 positive cells (n = 6).

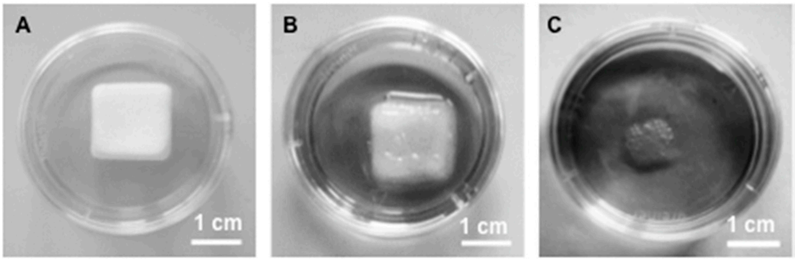

Figure S2. Evolution of WJ-MSCs-loaded HEMOCOLLAGENE® foam in culture. Photographs of HEMOCOLLAGENE® foam before (**A**) and after 4 (**B**) and 10 (**C**) days of immersion in culture media (scale bars indicate 1 cm).

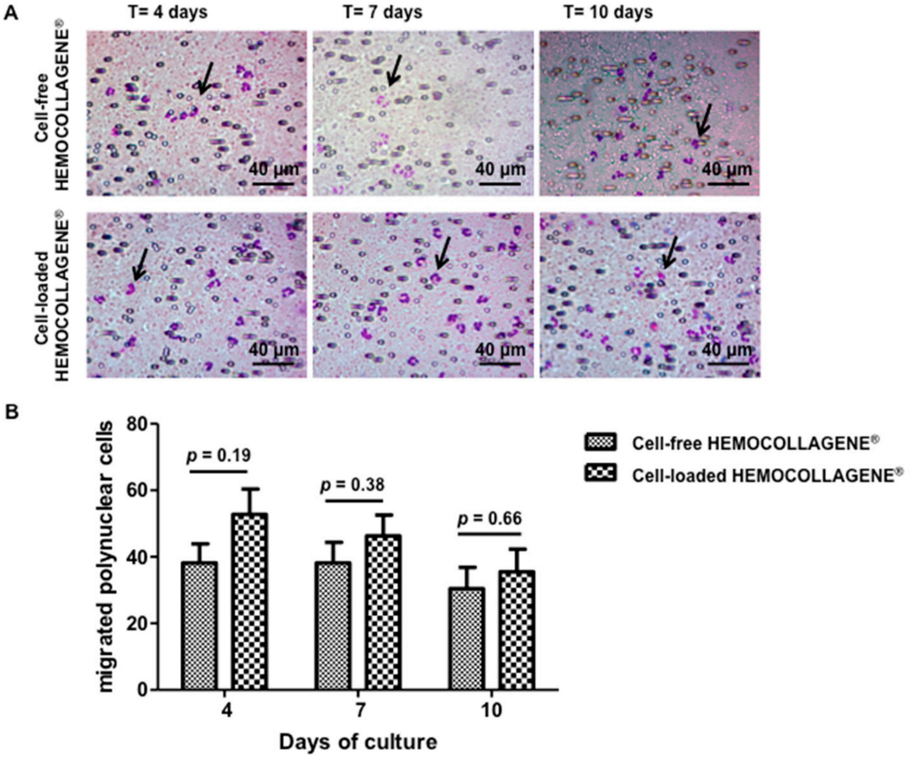

Figure S3. Neutrophils migration. (**A**) Representative pictures of insert membrane stained with May-Grünwald Giemsa showing migrated neutrophils attracted by WJ-MSCs-free HEMOCOLLAGENE® conditioned media (**upper** row) and by WJ-MSCs loaded-HEMOCOLLAGENE® conditioned media (**lower** row); black arrows, objective × 40, scale bars indicate 40 µm; (**B**) Histograms of quantified neutrophils, showing non-significant migration of neutrophils, whatever the tested condition (n = 6, Mann & Whitney statistical test).
